# Biological control of *Heterodera glycines* by spore-forming plant growth-promoting rhizobacteria (PGPR) on soybean

**DOI:** 10.1371/journal.pone.0181201

**Published:** 2017-07-13

**Authors:** Ni Xiang, Kathy S. Lawrence, Joseph W. Kloepper, Patricia A. Donald, John A. McInroy

**Affiliations:** Department of Entomology and Plant Pathology, Auburn University, Auburn, Alabama, United States of America; East Carolina University, UNITED STATES

## Abstract

*Heterodera glycines*, the soybean cyst nematode, is the most economically important plant-parasitic nematode on soybean production in the U.S. The objectives of this study were to evaluate the potential of plant growth-promoting rhizobacteria (PGPR) strains for mortality of *H*. *glycines* J2 *in vitro* and for reducing nematode population density on soybean in greenhouse, microplot, and field trials. The major group causing mortality to *H*. *glycines in vitro* was the genus *Bacillus* that consisted of 92.6% of the total 663 PGPR strains evaluated. The subsequent greenhouse, microplot, and field trials indicated that *B*. *velezensis* strain Bve2 consistently reduced *H*. *glycines* cyst population density at 60 DAP. *Bacillus mojavensis* strain Bmo3 suppressed *H*. *glycines* cyst and total *H*. *glycines* population density under greenhouse conditions. *Bacillus safensis* strain Bsa27 and Mixture 1 (Bve2 + Bal13) reduced *H*. *glycines* cyst population density at 60 DAP in the field trials. *Bacillus subtilis* subsp. *subtilis* strains Bsssu2 and Bsssu3, and *B*. *velezensis* strain Bve12 increased early soybean growth including plant height and plant biomass in the greenhouse trials. *Bacillus altitudinis* strain Bal13 increased early plant growth on soybean in the greenhouse and microplot trials. Mixture 2 (Abamectin + Bve2 + Bal13) increased early plant growth in the microplot trials at 60 DAP, and also enhanced soybean yield at harvest in the field trials. These results demonstrated that individual PGPR strains and mixtures can reduce *H*. *glycines* population density in the greenhouse, microplot, and field conditions, and increased yield of soybean.

## Introduction

*Heterodera glycines* Ichinohe, the soybean cyst nematode, was first reported in the United States in North Carolina in 1954 [1]. Now *H*. *glycines* has been found in every soybean-producing state in the U.S. except New York and West Virginia, due to their small soybean acreage and limited soybean production [2]. In the United States, *H*. *glycines* was the most important disease in soybean production, followed by *Phytophthora* root and stem rot and seedling diseases over the past 10 years [3]. Soybean yield losses caused by *H*. *glycines* were estimated to be 25% to 38% of total yield losses in 28 U.S. states, which is more than any other disease from 2006 to 2009 [4].

The removal of chemical nematicides such as Aldicarb (Temik) (Bayer CropScience, Raleigh, NC) has driven the investigation for alternative strategies for integrated pest management of plant-parasitic nematodes. Biological control agents previously assessed for the management of *H*. *glycines* were nematophagous fungi, endoparasitic fungi, female and egg-parasitic fungi, fungi producing antibiotic substances, vesicular-arbuscular mycorrhizal (VAM) fungi, *Pasteuria* spp., chitinolytic bacteria, and plant-growth-regulatory bacteria [5]. *Monacrosporium drechsleri*, an example of nematophagous fungi, has been found to attack J2 of *H*. *glycines* [6]. *Hirsutella rhossiliensis* and *H*. *minnesotensis*, are two endoparasitic fungi found to parasitize vermiform stages of *H*. *glycines* [7], and both were found highly effective against *H*. *glycines* through paratisizing J2 in the soil when applied at planting or two weeks prior to planting in the greenhouse [6]. The fungal genera *Exophiala*, *Fusarium*, *Gliocladium*, *Neocosmospora*, *Paecilomyces*, *Phoma*, *Stagonospora*, and *Pochonia* were commonly recovered from females and cysts of *H*. *glycines* [5]. Isolates from those fungi could be female and/or egg-parasitic fungi. Some fungi were found to produce antibiotic substance which inhibits eggs hatch or juvenile mobility. For example, an isolate of the fungus *Chaetomium globosum*, was found to produce a low molecular weight compound, flavipin, which inhibited *in vitro* egg hatch and juvenile mobility of *Meloidogyne incognita* and hatch of *H*. *glycines* [8]. VAM fungi were also reported to decrease numbers of *H*. *glycines*. Tylka et al. [9] found that numbers of *H*. *glycines* in roots and soil were decreased by VAM fungi by as much as 73% at the highest *H*. *glycines* inoculum level through 49 days after planting in the greenhouse experiments.

Bacteria are another large group that offered potential in reducing *H*. *glycines* population density. *Pasteuria* spp. was first reported to attack *H*. *elachista* in Japan in 1987 [10] and was later found to attack *H*. *glycines* in North America in 1994 [11]. Four chitinolytic bacterial strains were found to reduce numbers of *H*. *glycines* through the interaction with the chitin substrate mixed in the soil in the greenhouse [12]. Thirty-six of 201 rhizobacteria strains were also found to reduce numbers of soybean cysts, eggs, and J2 in the initial greenhouse tests [13]. Among 20 strains that suppressed (≥ 50%) *H*. *glycines* in the initial greenhouse screening test, four were *Pseudomonas* spp., two *Bacillus* spp. (*B*. *cereus* and *B*. *pumilus*), three *Paenibacillus* spp., and one *Streptomyces* spp. [13]. Plant-growth-regulatory bacteria especially plant-growth promoting rhizobacteria (PGPR) were found to have potential for the control of *H*. *glycines*. Kloepper et al. [14] found that *B*. *megaterium*, *B*. *pumilus*, and *Bacillus* spp. were antagonistic to *H*. *glycines* and *M*. *incognita*. Sharma [15] evaluated the efficiency of toxins from pure cultures of *B*. *sphaericus* (Bs 2362), *B*. *thuringiensis var*. *israelensis* (Bti-H-14), and *B*. *thuringiensis var*. *kurstaki* (Btk-HD-1) against *H*. *glycines* in a greenhouse pot experiment. However, none of the toxins significantly reduced the final nematode population density in relation to the untreated control. Sharma and Gomes [16] evaluated the effect of those toxins again on oviposition and J2 hatching of *H*. *glycines* race 3 in the greenhouse and found the number of hatched J2 treated with Bs 2362 was significantly less than the control in one experiment.

Among these antagonists, rhizobacteria, especially *Bacillus* PGPR, can promote plant growth and elicit significant reductions in the incidence or severity of various diseases on a diversity of hosts [17], and also elicit nematicidal activity or induced systemic resistance to plant-parasitic nematodes. Many of these species produce endospores which help the bacteria survive in a wide range of environmental conditions and have long-shelf life giving them an advantage as a commercial product. Some *Bacillus* strains have been developed into commercial products for plant disease and plant-parasitic nematode management, such as BioNem-WP/BioSafe (*B*. *firmus*) (AgroGreen, Israel) [18], BioYield (combination of *B*. *amyloliquefaciens* strain IN937a and *B*. *subtilis* strain GB03) (Gustafson LLC, USA) [17, 19], Nemix (*Bacillus* spp.) (AgriLife/Chr Hansen, Brazil) [20], VOTiVO (*B*. *firmus* GB-126) (Bayer CropScience, Germany) [21], and Pathway Consortia (mixture of *B*. *subtilis*, *B*. *licheniformis*, *B*. *megaterium*, *B*. *coagulans*, *Pseudomonas fluorescens*, *Streptomyces* spp., and *Trichoderma* spp.) (Pathway Holdings, USA) [22].

More research on beneficial PGPR strains as biocontrol agents for plant-parasitic nematodes management is needed. The overall objective of this project was to evaluate PGPR strains for biological control potential of *H*. *glycines* on soybean. The specific objectives were to assess the potential of PGPR strains for *H*. *glycines* J2 mortality percentage *in vitro* using high throughput screening and select strains to further test for *H*. *glycines* population density reduction and enhanced plant growth in the greenhouse, microplot, and field production systems.

## Materials and methods

### PGPR strains

A total of 663 PGPR strains were included in an *in vitro* study. These strains were originally isolated, identified, and maintained by J. W. Kloepper at Auburn University, Auburn, AL. Among these strains, 92.6% were *Bacillu*s spp. including 208 strains of *B*. *simplex*, 70 strains of *B*. *toyonensis*, 53 strains of *B*. *aryabhattai*, 51 strains of *B*. *cereus*, 44 strains of *B*. *mycoides*, 41 strains of *B*. *velezensis*, 35 strains of *B*. *safensis*, 21 strains of *B*. *altitudinis*, 21 strains of *B*. *weihenstephanensis*, 15 strains of *B*. *subtilis* subsp. *inaquosorum*, 13 strains of *B*. *methylotrophicus*, six strains of *B*. *pumilus*, five strains of *B*. *psychrosaccharolyticus*, four strains of each *B*. *mojavensis*, *B*. *subtilis* subsp. *subtilis*, and *B*. *thuringiensis*, three strains of *B*. *siamensis* and *B*. *tequilensis*, and 13 strains of other *Bacillus* spp. The remaining 8.4% of the strains, ten were *Sporosarcina globispora*, nine were *Paenibacillus amylolyticus*, four were *Paenibacillus lautus*, three were unknown species, and 23 were from multiple other genera. The PGPR strains, stored in 30% glycerol at -80°C, were transferred to tryptic soy agar (TSA) (VWR, Radnor, PA) plates, and incubated at 35°C for 24 hours. Vegetative cells of each strain were suspended in 5 ml of sterile distilled water in glass tubes. The concentration of bacterial vegetative cell suspensions was adjusted to 1 × 10^7^ CFU/ml.

### Nematode inoculum

The *H*. *glycines* used as inoculum *in vitro*, in the greenhouse and microplot experiments were from a culture maintained in the greenhouse since 2000. Eggs for the experiments were extracted from a 60-day-old soybean (“Asgrow 5935”, Monsanto, St. Louis, MO) stock culture maintained in 500 cm^3^ polystyrene pots. Soil was gently washed from the soybean roots and cysts and females were dislodged from the roots [23]. Water with the cyst and female suspension was poured through nested 850-μm-pore and 250-μm-pore sieves to separate trash from cysts and females [23]. Cysts and females were ground with a mortar and pestle to release the eggs. Eggs were washed with water and collected on a 25-μm-pore sieve and the suspension was centrifuged at 240 g for 1 minute using the sucrose centrifugation-flotation method [24]. For *in vitro* tests, *H*. *glycines* eggs were placed in a modified Baermann funnel [25] on a Slide Warmer (Model 77) (Marshall Scientific, Brentwood, NH) and incubated at 31°C for 5 to 7 days to obtain the J2 [26]. The J2 were collected on a 25-μm-pore sieve, transferred to 1.5 ml micro centrifuge tubes, centrifuged at 5,000 g for 1 minute, rinsed with sterile distilled water, and centrifuged at 5,000 g for 1 minute. The J2 suspensions were adjusted to 30 to 40 J2 per 10 μl of water [26, 27]. For greenhouse and microplot trials, eggs were enumerated at × 40 magnification with an inverted TS100 Nikon microscope and standardized to 2,000 eggs per cone-tainer for tests in the greenhouse or 50,000 eggs per pot for tests in the microplot [27].

### Tests *in vitro*

*In vitro* tests were conducted to assess mortality percentage of *H*. *glycines* J2 by PGPR strains. The PGPR vegetative cell suspensions and *H*. *glycines* J2 inocula were prepared as described previously. Ten μl of nematode suspension containing 30 to 40 *H*. *glycines* J2 were added in each well of a 100 μl 96-well plate. Ninety μl of each PGPR vegetative cell suspension was transferred into each test well of the 96-well plate. Clothianidin plus *B*. *firmus* I-1582 (Poncho/Votivo) (Bayer CropScience, Raleigh, NC) at a 0.7 μl / well (0.424 mg ai/seed), 100 million international unit (MIU) /well of *Pasteuria nishizawae* (Clariva) (Syngenta Greensboro, NC), and 1 granule/well of Aldicarb (Temik 15G) (Bayer CropScience, Raleigh, NC) were used as industry standards, and sterile distilled water was the untreated control. Each plate was sealed with parafilm (VWR, Radnor, PA) and incubated at room temperature for 48 hours. Numbers of live *H*. *glycines* J2 were enumerated and recorded at experiment initiation and 48 hours after exposure to the treatments. Viability of *H*. *glycines* J2 was determined using the sodium technique developed by Xiang and Lawrence [27] for high throughput screening of biological or chemical agents on plant-parasitic nematodes. Mortality percentage of *H*. *glycines* J2 were calculated as the following equation: [(live J2 prior to exposure − live J2 at 48 hours) / live J2 prior to exposure] × 100. Each bacterial treatment had four replications and the experiment was repeated.

### Plant material

The soybean (*Glycine max*) variety “Asgrow 5935” (Monsanto, St. Louis, MO) as reported by Monsanto to be susceptible to *H*. *glycines* was used for all the experiments.

### Trials in the greenhouse

Seventy two PGPR strains from the *in vitro* screenings with high J2 mortality were selected for initial evaluation in the greenhouse for their efficacy to reduce nematode population density and promote soybean plant growth. Confidential agreements were signed during this research study and only ten PGPR strains were available for further testing. These included *B*. *altitudinis* strains Bal11 and Bal13, *B*. *mojavensis* strain Bmo3, *B*. *safensis* strains Bsa26 and Bsa27, *B*. *subtilis* subsp. *subtilis* strains Bsssu2 and Bsssu3, *B*. *velezensis* strains Bve12 and Bve2, and *Fictibacillus solisalsi* strain Fso1. All the tests were conducted at the Plant Science Research Center (PSRC) located at Auburn University, Auburn, AL. Experiments were performed in 150 cm^3^ plastic cone-tainers (Stuewe & Sons Inc., Tangent, Oregon) filled with a soil:sand mix (60:40 v/v). The soil was a kalmia loamy sand (80% sand, 10% silt, and 10% clay) collected from Plant Breeding Unit (PBU) located at E.V. Smith Research Center of Auburn University near Tallassee, AL. Soil was steam pasteurized at 180°C for 60 minutes to 120 minutes and cooled for 24 hours. Steam pasteurizing process was repeated prior to use. Two soybean seeds were planted 2.5 cm deep in each cone-tainer. One ml of bacterial cell suspension (1×10^7^ CFU/ml) was inoculated on each seed at planting. For the nematicide controls, soybean seeds were treated with each compound following industrial recommendations: 0.13 mg a.i./seed of Clothianidin plus *B*. *firmus* I-1582 (Poncho/Votivo), or 0.15 mg a.i./seed of Abamectin (Avicta) (Syngenta, Greensboro, NC), or 10,000 million international unit (MIU) /ml of *Pasteuria nishizawae* (Clariva) (Syngenta Greensboro, NC) prior to planting. All seeds were treated with a Gustafson table-top seed treater (Bayer CropScience, Research Triangle Park, NC), mixed for 3 min in the 454-gm stainless steel bucket and allow to airdry before packaging [28]. One ml of tap water added to the seeds was used as the untreated control. One ml containing 2,000 *H*. *glycines* eggs was pipetted into each cone-tainer at planting. Experiments were arranged in a randomized complete block design (RCBD). Each treatment had five replications and the entire experiment was repeated twice. Soybean seedlings were thinned to one per cone-tainer after emergence. Plants were watered as needed. Supplemental light of 1000 watts halide bulbs producing 110,000 lumens was supplied to maintain the day length of 14 hours per day. Greenhouse temperature was ranged from 21°C to 35°C. Experiments were terminated at 60 DAP. Plant and nematode measurements were recorded. Plant measurements included Plant height (PH) and Biomass including shoot and root fresh weights (SFW/RFW). *Heterodera glycines* cyst and vermiform stage numbers were recorded. The *H*. *glycines* cysts were extracted from the soybean roots as described previously in inoculum preparation. Water suspension containing 150 cm^3^ of soil from cone-tainers was poured through nested 75-μm and 25-μm-pore sieve to extract vermiform stages (juveniles and males). Vermiform stages were collected on the 75-μm-pore sieve and centrifuged using sucrose centrifugation-flotation method [24].

### Trials in the microplot

The performance of five strains and two strain mixtures were evaluated for nematode population density, early growth promotion, and yield enhancement of soybean in the microplots. The strains included a strain of *B*. *altitudinis* (Bal13), a strain of *B*. *safensis* (Bsa27), a strain of *B*. *subtilis* subsp. *subtilis* (Bsssu2), two strains of *B*. *velezensis* (Bve12 and Bve2), and two mixtures Mixture 1 (Bve2 + Bal13) and Mixture 2 (seeds treated with Abamectin + Bve2 + Bal13). Mixtures were formed from the best performing strains based on greenhouse studies. The experiments were conducted at the PSRC. Experiments were established in 26.5 liter pots filled with a Kalmia loamy sand (80% sand, 10% silt, and 10% clay) collected from PBU. Nematodes were extracted from the non pasteurized soil as previously described and *H*. *glycines* population density was below the detection level of the extraction method descripted previously. Experiments were arranged in a RCBD with 6 replications for each treatment and the experiment was repeated twice. Ten soybean seeds were hand-planted at 2.5 cm in depth in a linear pattern to simulate a linear row foot in the field [27]. One ml bacterial suspension (1 × 10^7^ CFU/ml) was applied to each seed at planting. Five ml containing 50,000 *H*. *glycines* eggs were pipetted randomly in each pot at planting. Soybean seeds treated with Clothianidin plus *B*. *firmus* I-1582, Abamectin, and *P*. *nishizawae* as previously described were used as standards. The untreated control received 1 ml of tap water per seed. Each microplot received 30 ml per minute of water by an automatic drip irrigation system adjusted throughout the season to run for 15–45 minutes twice a day, for a total of 450–1350 ml of water per microplot per day. At 60 DAP, one representative soybean plant was dug from each microplot for PH and Biomass (SFW + RFW) measurements and nematode extraction as previously described. Cysts were extracted from the roots. Vermiform stages were extracted from 100 cm^3^ of soil surrounding the roots. Total nematode numbers including cysts and vermiforms were recorded. At plant maturity, approximately 160 DAP, soybeans were harvested and yield was recorded as grams of soybean seed per plot.

### Trials in the field

The same strains and mixtures assessed in the microplot trials were evaluated in field trials for their effect on early-season nematode population density, plant growth promotion, and yield enhancement in soybean. The experiments were established in the research stations of E.V. Smith in a Wickham fine sandy loam soil (70% sand, 16% silt, and 18% clay), Tallassee, AL and Tennessee Valley Research and Extension Center (TVREC) in a Decatur silt loam soil (24% sand, 49% silt, and 28% clay), Belle Mina, AL. Both were artificially infested fields with soybean cysts added every year since 2011. The experiments were arranged in a RCBD with 5 replications for each treatment. The field trials were arranged in two-row plots that were 7 m long with 0.9 m row spacing. Blocks were separated by a 6 m alley. One hundred and seventy five soybean seeds were planted in each row with an Almaco plot planter (Almaco, Iowa). The PGPR treatments were applied as in-furrow spray standardized to 1×10^7^ CFU/seed and applied at 32.5 liter per hectare at planting. Seeds treated with Clothianidin plus *B*. *firmus* I-1582, Abamectin, and *P*. *nishizawae* as previously described were included as industry standard controls. Tap water applied in-furrow was used as untreated control. At 60 DAP, four random soybean plants were removed from each plot. The same plant growth parameters evaluated in the microplots were evaluated in the field. *Heterodera glycines* population density was determined by extracting soybean cysts and females from the roots, and vermiform stages from the soil as described previously. Soybeans were harvested mechanically with a Almaco plot harvester (Almaco, Iowa) at plant maturity approximately 160 DAP and yield recorded and adjusted to 13% moisture content.

### Statistical analysis

Data collected from *in vitro*, greenhouse, microplot, and field trials were analyzed in SAS 9.4 (SAS Institute, Cary, NC) using the PROC GLIMMIX procedure. Dependent variables included J2 mortality, plant height (PH), biomass (Bio), cyst, vermiform stage (VS), total SCN, and yield. Fixed effects were PGPR strains or nematicides treatments and the random effects included replication, repeat in time, and location. Student panels were generated to determine the normality of the residuals. A log-normal distribution transformation was required for the PH, Bio, cyst, VS, total SCN, and yield data to satisfy the normal assumptions. LS-means were compared between the treatments, chemical standards Clothianidin plus *B*. *firmus* I-1582, Abamectin, *P*. *nishizawae* and the untreated control by Dunnett’s method at significant level of *P* ≤ 0.05 or *P* ≤ 0.10. The LS-means are presented in the tables with adjusted *P* values for statistical differences.

## Results

### Test *in vitro*

The mortality percentage of *H*. *glycines* J2 ranged from 0.0% to 99.9% with the PGPR strains tested (663) with an average of 16.0%. Data presented were results of 52 strains LS-means greater than 50% mortality of *H*. *glycines* J2 (Table 1). The PROC GLIMMIX analysis indicated the numerator and denominator df are 666 and 1875, respectively with an F value of 8.01, and *P* < 0.0001. Of those 52 strains, 24 were *B*. *simplex*, five were *B*. *altitudinis*, five were *B*. *toyonensis*, three were *B*. *aryabhattai*, three were *B*. *safensis*, two were *B*. *mycoides*, two were *B*. *subtilis* subsp. *subtilis*, and the remaining were *B*. *lentus*, *B*. *methylotrophicus*, *B*. *mojavensis*, *B*. *pumilus*, *B*. *weihenstephanensis*, *Fictibacillus solisalsi*, *Paenibacillus taichungensis*, and *P*. *xylanexedens*. Among all the PGPR strains tested, 6.8% caused significantly greater level of mortality percentage than the biological standard Clothianidin plus *B*. *firmus* I-1582 (*P* ≤ 0.05); 7.9% caused significantly greater level of mortality percentage than the level caused by *P*. *nishizawae* (*P* ≤ 0.05); 5.6% caused statistically similar mortality percentage to the level caused by Aldicarb (*P* ≤ 0.05); and 13.2% caused significantly greater mortality percentage than the level caused by untreated control (*P* ≤ 0.05) (Table 1). Among all the strains, 92.6% were *Bacillus* spp. strains, which was the major genera with greater mortality percentage than any other single genera.

**Table 1:** PGPR strains effect on *Heterodera glycines* J2 with LS-means more than 50% mortality^a^.

Code	Scientific name	*Heterodera glycines*	Dunnett's *P* vs^d^ (*P* ≤ 0.05)			
		J2 mortality (%)^b^	Clothianidin	*P*. *nishizawae*	Aldicarb	Water
			*+ B*. *firmus*^c^			
Bal9	*Bacillus altitudinis*	51.7	0.1099	0.0206	<.0001	<.0001
Bal11	*Bacillus altitudinis*	64.0	0.0236	0.0045	0.1725	<.0001
Bal12	*Bacillus altitudinis*	54.7	0.0408	0.0059	0.0002	<.0001
Bal13	*Bacillus altitudinis*	81.2	<.0001	<.0001	1.0000	<.0001
Bal20	*Bacillus altitudinis*	55.1	0.0353	0.0050	0.0003	<.0001
Bar15	*Bacillus aryabhattai*	90.5	<.0001	<.0001	1.0000	<.0001
Bar16	*Bacillus aryabhattai*	64.9	0.0180	0.0033	0.2079	<.0001
Bar21	*Bacillus aryabhattai*	57.5	0.0136	0.0016	0.0011	<.0001
Ble1	*Bacillus lentus*	74.2	<.0001	<.0001	0.4208	<.0001
Bmo3	*Bacillus mojavensis*	54.5	0.2720	0.0907	0.0117	0.0010
Bmt10	*Bacillus methylotrophicus*	51.4	0.4749	0.1896	0.0039	0.0033
Bmy19	*Bacillus mycoides*	66.9	0.0092	0.0015	0.3115	<.0001
Bmy32	*Bacillus mycoides*	77.7	0.0001	<.0001	0.9947	<.0001
Bpu6	*Bacillus pumilus*	78.4	<.0001	<.0001	0.9982	<.0001
Bsa25	*Bacillus safensis*	62.5	0.0378	0.0079	0.1200	<.0001
Bsa26	*Bacillus safensis*	74.1	0.0006	<.0001	0.8614	<.0001
Bsa27	*Bacillus safensis*	79.2	<.0001	<.0001	0.9997	<.0001
Bsp2	*Bacillus simplex*	60.2	0.0044	0.0004	0.0038	<.0001
Bsp3	*Bacillus simplex*	62.0	0.0437	0.0095	0.1061	<.0001
Bsp4	*Bacillus simplex*	93.9	<.0001	<.0001	1.0000	<.0001
Bsp8	*Bacillus simplex*	55.9	0.2035	0.0626	0.0186	0.0005
Bsp26	*Bacillus simplex*	64.5	0.0201	0.0038	0.1927	<.0001
Bsp53	*Bacillus simplex*	81.9	<.0001	<.0001	1.0000	<.0001
Bsp68	*Bacillus simplex*	87.1	<.0001	<.0001	1.0000	<.0001
Bsp90	*Bacillus simplex*	52.2	0.0340	0.0038	<.0001	<.0001
Bsp113	*Bacillus simplex*	63.3	0.0010	<.0001	0.0144	<.0001
Bsp123	*Bacillus simplex*	74.2	0.0005	<.0001	0.8715	<.0001
Bsp129	*Bacillus simplex*	99.9	<.0001	<.0001	1.0000	<.0001
Bsp130	*Bacillus simplex*	61.6	0.0490	0.0109	0.0960	<.0001
Bsp133	*Bacillus simplex*	73.7	0.0007	<.0001	0.8329	<.0001
Bsp139	*Bacillus simplex*	67.6	0.0072	0.0011	0.3548	<.0001
Bsp141	*Bacillus simplex*	99.9	<.0001	<.0001	1.0000	<.0001
Bsp146	*Bacillus simplex*	70.9	0.0021	0.0003	0.6075	<.0001
Bsp149	*Bacillus simplex*	64.7	0.0189	0.0035	0.2013	<.0001
Bsp153	*Bacillus simplex*	89.7	<.0001	<.0001	1.0000	<.0001
Bsp159	*Bacillus simplex*	56.8	0.1650	0.0480	0.0251	0.0004
Bsp165	*Bacillus simplex*	71.4	<.0001	<.0001	0.2188	<.0001
Bsp168	*Bacillus simplex*	69.1	0.0042	0.0006	0.4596	<.0001
Bsp171	*Bacillus simplex*	67.3	0.0079	0.0013	0.3390	<.0001
Bsp188	*Bacillus simplex*	73.0	0.0009	0.0001	0.7829	<.0001
Bsp196	*Bacillus simplex*	95.1	<.0001	<.0001	1.0000	<.0001
Bsssu2	*Bacillus subtilis* subsp. *subtilis*	74.8	0.0004	<.0001	0.9084	<.0001
Bsssu3	*Bacillus subtilis* subsp. *subtilis*	74.2	0.0005	<.0001	0.8715	<.0001
Bto10	*Bacillus toyonensis*	64.7	0.0005	<.0001	0.0250	<.0001
Bto11	*Bacillus toyonensis*	62.7	0.0013	0.0001	0.0114	<.0001
Bto22	*Bacillus toyonensis*	64.8	0.0004	<.0001	0.0265	<.0001
Bto23	*Bacillus toyonensis*	51.1	0.1304	0.0258	<.0001	<.0001
Bto51	*Bacillus toyonensis*	67.6	<.0001	<.0001	0.0718	<.0001
Bve2	*Bacillus velezensis*	54.7	0.2613	0.0861	0.0125	0.0009
Bwe6	*Bacillus weihenstephanensis*	93.3	<.0001	<.0001	1.0000	<.0001
Fso1	*Fictibacillus solisalsi*	59.6	0.0834	0.0206	0.0572	0.0001
Pata1	*Paenibacillus taichungensis*	64.4	0.0211	0.0040	0.1865	<.0001
Paxy1	*Paenibacillus xylanexedens*	74.8	<.0001	<.0001	0.4681	<.0001
**Control**	**Active ingredient**^c^					
Poncho/Votivo	Clothianidin	21.1	…	1.0000	<.0001	0.9885
	*+B*. *firmus* I-1582					
Clariva	*Pasteuria nishizawae*	16.3	1.0000	…	<.0001	0.0000
Temik	Aldicarb	99.6	<.0001	<.0001	…	<.0001
Untreated control	Sterile distilled water	2.8	0.9885	1.0000	<.0001	…

*In vitro* tests were performed in 96-well plates. Data collected were analyzed in SAS 9.4 using PROC GLIMMIX procedure at significantlevel of α ≤ 0.05. *P* value less than 0.05 indicate a significant effect. Adjusted *P* values were obtained according to Dunnett's method.

^a^The LS-means are presented in the tables with adjusted *P* values for statistical differences.

^b^Mortality percentage was determined by calculating as the following equation: [(live J2 prior to exposure − live J2 at 48 hours) / live J2 prior to exposure] × 100.

^c^Active ingredients for the nematicides Poncho/Votivo are Clothianidin plus *B*. *firmus* I-1582, Clariva is *Pasteuria nishizawae*, Temik is Aldicarb, and untreated control is sterile distilled water.

^d^Dunnett's option was used in the LSMEANS statement to assess the differences between bacterial strains and the Poncho/Votivo, Clariva, Temik, and the untreted control.

### Greenhouse trial

**Fig 1:**
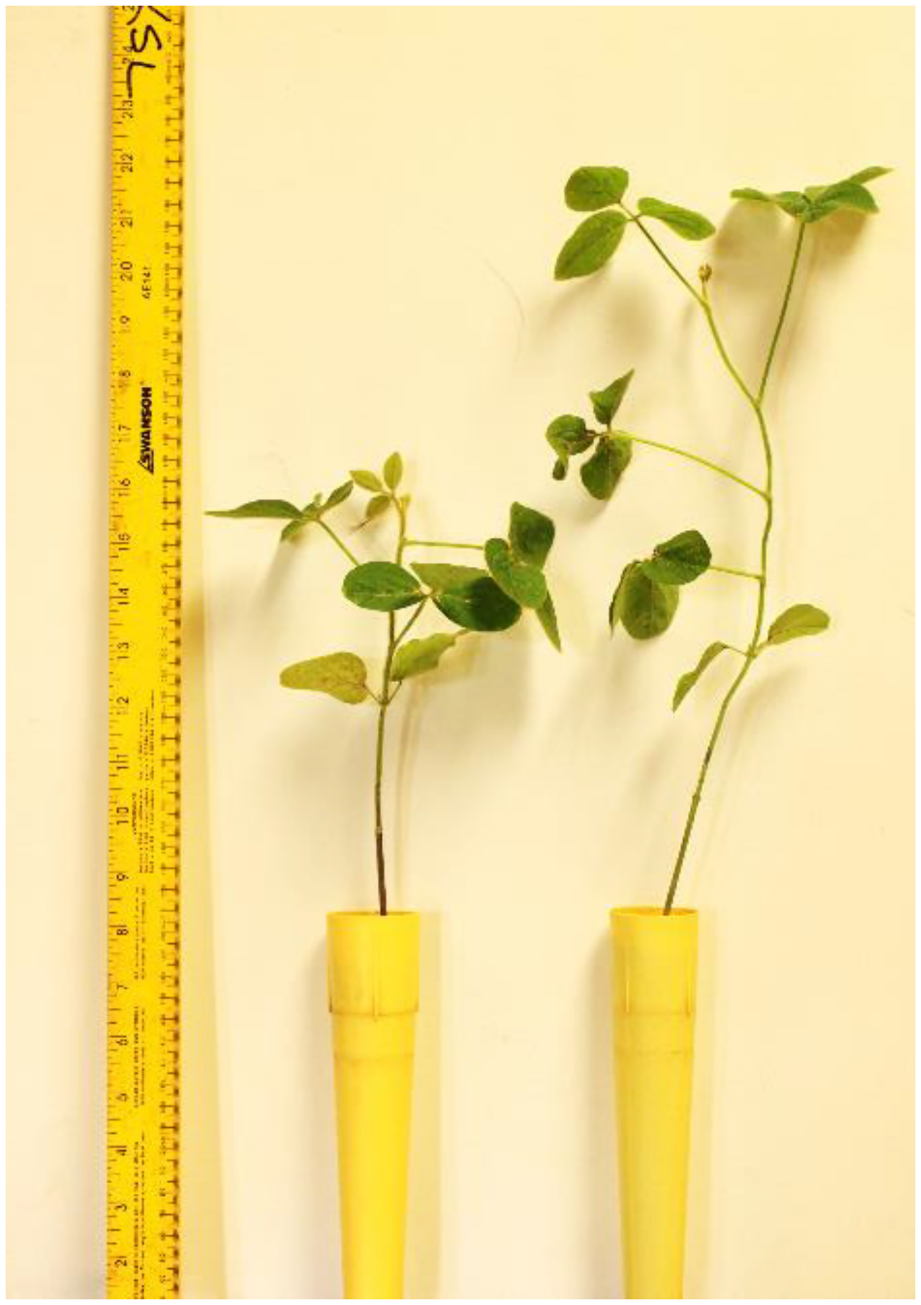
Soybean plants treated with strain *B*. *altitudinis* Bal13 (Right) and untreated control (Left) at 60 DAP.

**Fig 2:**
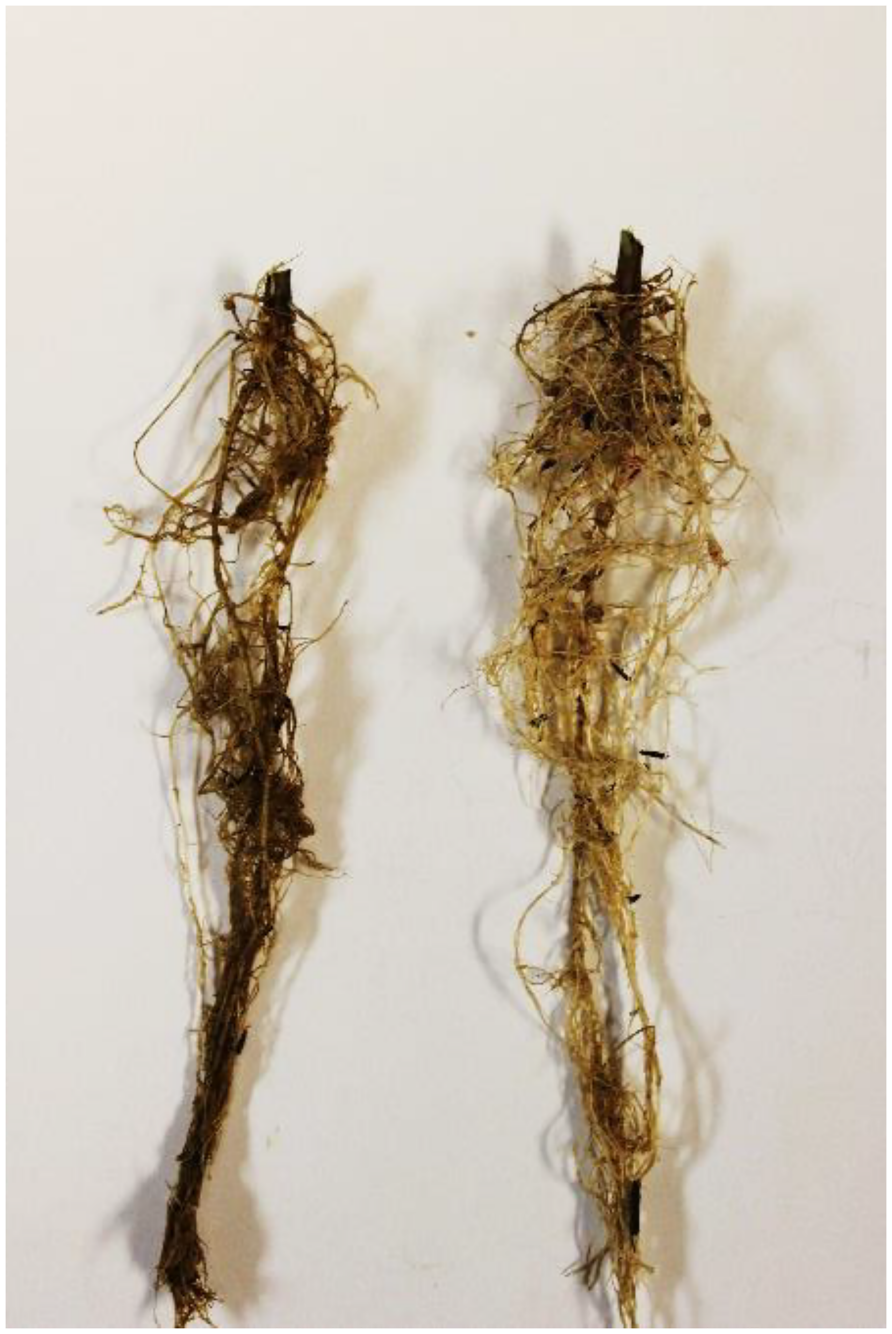
Soybean roots treated with strain *B*. *altitudinis* Bal13 (Right) and untreated control (Left) at 60 DAP.

The PROC GLIMMIX analysis for the greenhouse trials indicated the numerator and denominator df are 13 and 117, respectively with an F value of 2.34, and *P* = 0.0083. Strains *B*. *mojavensis* Bmo3 and *B*. *velezensis* Bve2 suppressed *H*. *glycines* cyst population density at 60 DAP at levels statistically equivalent to Abamectin (*P* ≤ 0.10) (Table 2). Strains *B*. *mojavensis* Bmo3, *B*. *subtilis* subsp. *subtilis* Bsssu2, *B*. *velezensis* Bve2, and *Fictibacillus solisalsi* Fso1 suppressed total *H*. *glycines* including cysts and vermiform stages at 60 DAP at levels statistically equivalent to Abamectin (*P* ≤ 0.10) (Table 2). All ten PGPR strains significantly increased the soybean plant height compared to the standard Clothianidin plus *B*. *firmus* I-1582 at 60 DAP (*P* ≤ 0.05) (Table 3). Strains *B*. *altitudinis* Bal13 (Figs 1 and 2), *B*. *subtilis* subsp. *subtilis* Bsssu2 and Bsssu3, and *B*. *velezensis* Bve12 significantly increased plant biomass (SFW + RFW) compared to the standard Clothianidin plus *B*. *firmus* I-1582 at 60 DAP (*P* ≤ 0.05) (Table 3).

**Table 2:** Effect of ten PGPR strains on *Heterodera glycines* cyst numbers and total nematode population density in greenhouse trials at 60 DAP^a^.

Treatment	Scientific Name	Cyst^b^		60 DAP					60 DAP		
				Dunnett's *P* vs. (*P* ≤ 0.10)					Dunnett's *P* vs. (*P* ≤ 0.10)		
			Clothianidin	*P*. *nishizawae*	Abamectin	Water	Total *H*. *glycines*^d^	Clothianidin	*P*. *nishizawae*	Abamectin	Water
			*+ B*. *firmus*^c^					+ *B*. *firmus*			
Bal11	*B*. *altitudinis*	2458	0.9599	1.0000	0.0400	1.0000	2897	1.0000	1.0000	0.0876	1.0000
Bal13	*B*. *altitudinis*	2154	0.9860	1.0000	0.0556	1.0000	3817	0.9781	0.9939	0.0187	1.0000
Bmo3	*B*. *mojavensis*	1665	1.0000	0.9931	0.2698	0.9678	2319	1.0000	1.0000	0.2928	0.9900
Bsa26	*B*. *safensis*	2934	0.6536	0.9993	0.0092	1.0000	3781	0.9840	0.9960	0.0206	1.0000
Bsa27	*B*. *safensis*	2754	0.9449	1.0000	0.0353	1.0000	3132	1.0000	1.0000	0.0893	1.0000
Bsssu2	*B*. *subtilis* subsp. *subtilis*	2140	0.9940	1.0000	0.0759	1.0000	2474	1.0000	1.0000	0.1558	1.0000
Bsssu3	*B*. *subtilis* subsp. *subtilis*	2064	0.8248	1.0000	0.0184	1.0000	2780	0.9966	0.9995	0.0306	1.0000
Bve2	*B*. *velezensis*	1583	1.0000	0.9780	0.3331	0.9282	1822	0.9966	0.9859	0.5600	0.8386
Bve12	*B*. *velezensis*	3527	0.3012	0.9062	0.0018	0.9644	4197	0.7629	0.8500	0.0047	0.9865
Fso1	*Fictibacillus solisalsi*	1733	0.9991	1.0000	0.0944	1.0000	2326	1.0000	1.0000	0.1187	1.0000
**Control**	**Active ingredient**^c^										
Poncho/Votivo	Clothianidin	1745	…	0.9832	0.3554	0.9424	2386	…	1.0000	0.1875	0.9999
	*+B*. *firmus* I-1582										
Clariva	*Pasteuria nishizawae*	2245	0.9832	…	0.0594	1.0000	2562	1.0000	…	0.1446	1.0000
Avicta	Abamectin	1116	0.3715	0.0620	…	0.0352	1789	0.1963	0.1513	…	0.0562
Untreated control	Water	2304	0.9343	1.0000	0.0327	…	3274	0.9999	1.0000	0.0520	…

Greenhouse trials were performed in plastic cone-tainers with mixed pasteurized soil and sand (60:40 v/v) for 45 days. Data collected were repeated twice and analyzed in SAS 9.4 using PROC GLIMMIX procedure at significant level of α ≤ 0.10. Adjusted *P* values less than 0.10 indicated a significant effect. Adjusted *P* values were obtained by analyzing data according to Dunnett’s method.

^a^The LS-means and adjusted *P* values are presented in the tables.

^b^Cyst = soybean cysts and white females at 60 DAP.

^c^Active ingredients for the nematicides Poncho/Votivo are Clothianidin plus *B*. *firmus* I-1582, Clariva is *Pasteuria nishizawae*, Avicta is Abamectin, and untreated control is water.

^d^Total *H*. *glycines* = total numbers of soybean cysts, white females, and juveniles at 60 DAP.

**Table 3:** Effect of ten PGPR strains on soybean plant height and plant biomass in greenhouse trials at 60 DAP^a^.

Treatment	Scientific Name	PH^b^		60 DAP			Bio^d^		60 DAP		
				Dunnett's *P* vs. (*P* ≤ 0.05)					Dunnett's *P* vs. (*P* ≤ 0.05)		
			Clothianidin	*P*. *nishizawae*	Abamectin	Water		Clothianidin	*P*. *nishizawae*	Abamectin	Water
			+ *B*. *firmus*^c^					+ *B*. *firmus*			
Bal11	*B*. *altitudinis*	35.3	0.0164	1.0000	0.9971	1.0000	4.9	0.1865	0.9910	0.9971	0.9845
Bal13	*B*. *altitudinis*	35.1	0.0154	1.0000	0.9962	1.0000	5.4	0.0116	1.0000	1.0000	1.0000
Bmo3	*B*. *mojavensis*	40.8	0.0002	0.6444	0.3767	0.9827	4.6	0.1871	0.9909	0.9970	0.9842
Bsa26	*B*. *safensis*	38.9	0.0014	0.9352	0.6983	1.0000	4.8	0.0566	1.0000	1.0000	1.0000
Bsa27	*B*. *safensis*	35.4	0.0109	1.0000	0.9870	1.0000	4.8	0.0766	1.0000	1.0000	1.0000
Bsssu2	*B*. *subtilis* subsp. *subtilis*	41.4	0.0002	0.4976	0.2746	0.9227	5.4	0.0319	1.0000	1.0000	1.0000
Bsssu3	*B*. *subtilis* subsp. *subtilis*	34.9	0.0255	1.0000	0.9997	0.9999	5.2	0.0399	1.0000	1.0000	1.0000
Bve2	*B*. *velezensis*	34.7	0.0279	1.0000	0.9998	0.9998	5.3	0.0771	1.0000	1.0000	1.0000
Bve12	*B*. *velezensis*	37.9	0.0020	0.9654	0.7667	1.0000	6.1	0.0028	0.9972	0.9921	0.9986
Fso1	*Fictibacillus solisalsi*	35.0	0.0187	1.0000	0.9984	1.0000	4.3	0.1577	0.9963	0.9990	0.9930
**Control**	**Active ingredient**^c^										
Poncho/Votivo	Clothianidin	27.1	…	0.0477	0.1414	0.0058	2.8	…	0.0319	0.0495	0.0227
	*+B*. *firmus* I-1582										
Clariva	*Pasteuria nishizawae*	34.2	0.0477	…	1.0000	0.9990	5.0	0.0319	…	1.0000	1.0000
Avicta	Abamectin	33.7	0.1481	1.0000	…	0.9546	5.2	0.0517	1.0000	…	1.0000
Untreated control	Water	37.6	0.0056	0.9988	0.9385	…	5.3	0.0220	1.0000	1.0000	…

Greenhouse trials were performed in plastic cone-tainers with mixed pasteurized soil and sand (60:40 v/v) for 60 days. Data collected were repeated twice and analyzed in SAS 9.4 using PROC GLIMMIX procedure at significant level of 0.05. Adjusted *P* values less than 0.05 indicated a significant effect. Adjusted *P* values were obtained by analyzing data according to Dunnett’s method.

^a^The LS-means are presented in the tables with adjusted *P* values for statistical differences.

^b^PH = plant height (cm) at 60 DAP.

^c^Active ingredients for the nematicides Poncho/Votivo are Clothianidin plus *B*. *firmus* I-1582, Clariva is *Pasteuria nishizawae*, Avicta is Abamectin, and untreated control is water.

^d^Bio = soybean plant biomass including shoot fresh weight (g) and root fresh weight (g) at 60 DAP.

### Microplot trial

Five *Bacillus* PGPR strains and two mixtures were evaluated in the microplot for early plant growth promotion, reduction of *H*. *glycines* population density, and yield enhancement. The PROC GLIMMIX analysis for the microplot trials indicated the numerator and denominator df are 10 and 90, respectively with an F value of 2.60, and *P* = 0.0080. Results indicated that the *B*. *velezensis* strain Bve2 significantly reduced *H*. *glycines* cyst numbers compared to the biological standard *P*. *nishizawae* at 60 DAP (*P* ≤ 0.10) (Table 4). *Bacillus altitudinis* strain Bal13 and Mixture 2 significantly increased plant height compared to all the industrial standards (*P* ≤ 0.10) (Table 5). *Bacillus altitudinis* strain Bal13, *B*. *safensis* strain Bsa27, and Mixture 2 significantly increased plant biomass (SFW + RFW) compared to the untreated control at 60 DAP (*P* ≤ 0.10) (Table 5). Number of *H*. *glycines* vermiform stage (data not show) at 60 DAP and soybean yield (Table 5) at harvest were similar among all the PGPR strains and the industrial standards.

**Table 4:** Effect of five PGPR strains and two mixtures of PGPR strains on *Heterodera glycines* population density on soybean in microplot trials at 60 DAP^a^.

Treatment	Scientific Name	Cyst^b^		60 DAP					60 DAP		
				Dunnett's *P* vs. (*P* ≤ 0.10)					Dunnett's *P* vs. (*P* ≤ 0.10)		
			Clothianidin	*P*. *nishizawae*	Abamectin	Water	Total *H*. *glycines*^d^	Clothianidin	*P*. *nishizawae*	Abamectin	Water
			*+ B*. *firmus*^c^					*+ B*. *firmus*			
Bal13	*B*. *altitudinis*	1123	0.0449	0.6546	0.0611	0.1065	1224	0.0791	0.8444	0.1114	0.2987
Bsa27	*B*. *safensis*	472	0.9998	0.3982	0.9986	0.9833	609	1.0000	0.7686	1.0000	0.9995
Bsssu2	*B*. *subtilis* subsp. *subtilis*	774	0.7977	1.0000	0.8814	0.9752	984	0.3261	1.0000	0.4340	0.8383
Bve12	*B*. *velezensis*	439	0.9899	0.1373	0.9678	0.8624	448	0.9960	0.1455	0.9793	0.7078
Bve2	*B*. *velezensis*	384	0.9042	0.0627	0.8277	0.6375	425	0.9875	0.1131	0.9543	0.6243
Mixture 1^e^		465	0.9996	0.3750	0.9977	0.9776	471	0.9998	0.3643	0.9980	0.9041
Mixture 2^e^		930	0.4621	0.9997	0.4589	0.6263	968	0.5817	1.0000	0.6898	0.9537
**Control**	**Active ingredient**^c^										
Poncho/Votivo	Clothianidin	563	…	0.5400	1.0000	0.9999	584	…	0.4944	1.0000	0.9914
	*+B*. *firmus* I-1582										
Clariva	*Pasteuria nishizawae*	832	0.5400	…	0.6467	0.7878	931	0.4944	…	0.6216	0.9537
Avicta	Abamectin	587	1.0000	0.6467	…	1.0000	620	1.0000	0.6216	…	0.9989
Untreated control	Water	632	0.9999	0.8361	1.0000	…	736	0.9914	0.9539	0.9989	…

Microplot trials were performed in 26.5 liter pot. Data collected were repeated and analyzed in SAS 9.4 using PROC GLIMMIX procedure at significant level of α ≤ 0.10. Adjusted *P* values less than 0.10 indicated a significant effect. Adjusted *P* values were obtained by analyzing data according to Dunnett’s method.

^a^The LS-means are presented in the tables with adjusted *P* values for statistical differences.

^b^Cyst = cysts and white females from 100 cm^3^ of soil at 60 DAP.

^c^Active ingredients for the nematicides Poncho/Votivo are Clothianidin plus *B*. *firmus* I-1582, Clariva is *Pasteuria nishizawae*, Avicta is Abamectin, and untreated control is water.

^d^Total *H*. *glycines* = total numbers of soybean cysts, white females, and vermiform stages per 100 cm^3^ of soil at 60 DAP.

^e^Mixture 1 = strain Bve2 + strain Bal13; Mixture 2 = Abamectin + strain Bve2 + strain Bal13.

**Table 5:** Effect of five PGPR strains and two mixtures of PGPR strains on early plant growth at 60 DAP and yield on soybean at 160 DAP in the microplot^a^.

Treatment	Scientific Name	PH^b^		60 DAP			Bio^d^		60 DAP			Yield^e^		160 DAP		
				Dunnett's *P* vs. (*P* ≤ 0.10)					Dunnett's *P* vs. (*P* ≤ 0.10)					Dunnett's *P* vs. (*P* ≤ 0.10)		
			Clothianidin	*P*. *nishizawae*	Abamectin	Water		Clothianidin	*P*. *nishizawae*	Abamectin	Water		Clothianidin	*P*. *nishizawae*	Abamectin	Water
			*+ B*. *firmus*^c^					*+ B*. *firmus*					*+ B*. *firmus*			
Bal13	*B*. *altitudinis*	43.8	0.0476	0.0689	0.0938	0.0389	95.7	0.1523	0.1388	0.4995	0.0184	192.2	1.0000	0.9999	0.9748	0.9974
Bsa27	*B*. *safensis*	41.7	0.1396	0.1903	0.2450	0.1176	94.7	0.1308	0.1189	0.4508	0.0150	175.3	0.9998	1.0000	0.7707	1.0000
Bsssu2	*B*. *subtilis* subsp. *subtilis*	36.1	0.9462	0.9842	0.9965	0.9120	73.6	0.7390	0.7027	0.9986	0.1484	193.2	0.9996	0.9708	1.0000	0.8941
Bve12	*B*. *velezensis*	38.4	0.4498	0.5745	0.6872	0.3893	66.0	0.9773	0.9672	1.0000	0.3954	203.0	0.9997	0.9758	0.9999	0.9056
Bve2	*B*. *velezensis*	36.7	0.7664	0.8727	0.9388	0.7015	76.7	0.7185	0.6818	0.9979	0.1394	219.1	0.9782	0.8365	1.0000	0.6875
Mixture 1^f^		39.8	0.3309	0.4216	0.5098	0.2884	74.4	0.7053	0.6742	0.9898	0.1880	156.3	0.8853	0.9900	0.3279	0.9994
Mixture 2^f^		43.8	0.0478	0.0691	0.0940	0.0390	88.5	0.4328	0.4048	0.8835	0.0812	185.4	1.0000	0.9993	0.9925	0.9885
**Control**	**Active ingredient**^c^															
Poncho/Votivo	Clothianidin	33.3	…	1.0000	1.0000	1.0000	57.4	…	1.0000	0.9880	0.9435	181.6	…	1.0000	0.9718	0.9979
	*+B*. *firmus* I-1582															
Clariva	*Pasteuria nishizawae*	33.6	1.0000	…	1.0000	1.0000	53.4	1.0000	…	0.9815	0.9585	178.9	1.0000	…	0.8163	1.0000
Avicta	Abamectin	34.2	1.0000	1.0000	…	0.9999	66.5	0.9880	0.9815	…	0.4460	204.6	0.9718	0.8163	…	0.6634
Untreated control	Water	32.9	1.0000	1.0000	0.9999	…	48.9	0.9435	0.9585	0.4460	…	160.8	0.9979	1.0000	0.6634	…

Microplot trials were performed in 26.5 liter pot. Data collected were repeated and analyzed in SAS 9.4 using PROC GLIMMIX procedure at significant level of α ≤ 0.10. Adjusted *P* values less than 0.10 indicated a significant effect. Adjusted *P* values were analyzed according to Dunnett’s method.

^a^The LS-means are presented in the tables with adjusted *P* values for statistical differences.

^b^PH = plant height (cm) at 60 DAP.

^c^Activie ingredients for the nematicides Poncho/Votivo are Clothianidin plus *B*. *firmus* I-1582, Clariva is *Pasteuria nishizawae*, Avicta is Abamectin, and untreated control is water.

^d^Bio = plant biomass including shoot fresh weight and root fresh weight (g) at 60 DAP.

^e^Yield = soybean yield (g) obtained at 160 DAP and adjusted to 13% moisture content per pot.

^f^Mixture 1 = strain Bve2 + strain Bal13; Mixture 2 = Abamectin + strain Bve2+ strain Bal13.

### Field trial

**Fig 3:**
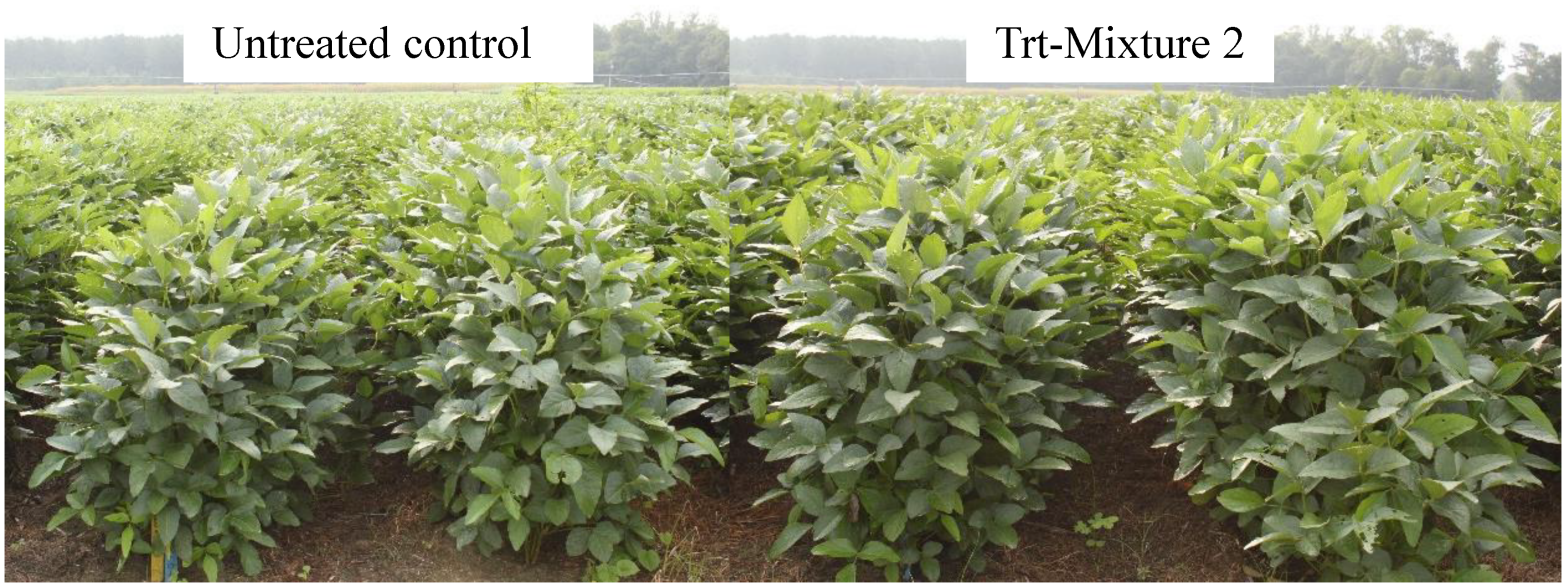
Soybean treated with Mixture 2 = Abamectin + strain Bve2 + strain Ball3 (Right) and untreated control (Left) at 80 DAP.

The PROC GLIMMIX analysis for the field trials indicated the numerator and denominator df are 10 and 71, respectively with an F value of 2.19, and *P* = 0.0280. Strains *B*. *safensis* Bsa27, *B*. *velezensis* Bve2, and Mixture 1 significantly reduced *H*. *glycines* cyst numbers compared to untreated control at 60 DAP (*P* ≤ 0.10) (Table 6). Strain Mixture 2 (Fig 3) significantly increased soybean yield compared to the untreated control at 160 DAP (*P* ≤ 0.10) (Table 6). Plant height, biomass, *H*. *glycines* vermiform stages, and total *H*. *glycines* were similar among all the PGPR strains and industrial standards (data not show).

**Table 6:** Effects of five PGPR strains and two mixtures of PGPR strains on early soybean plant growth and yield in the field trials^a^.

Treatment	Scientific Name	Bio^b^		60 DAP			Cyst^d^		60 DAP					160 DAP		
				Dunnett's *P* vs. (*P* ≤ 0.10)					Dunnett's *P* vs. (*P* ≤ 0.10)					Dunnett's *P* vs. (*P* ≤ 0.10)		
			Clothianidin	*P*. *nishizawae*	Abamectin	Water		Clothianidin	*P*. *nishizawae*	Abamectin	Water	Yield^e^	Clothianidin	*P*. *nishizawae*	Abamectin	Water
			*+B*. *firmus*^c^					*+B*. *firmus*					*+ B*. *firmus*			
Bal13	*B*. *altitudinis*	70.1	1.0000	0.9993	1.0000	1.0000	136	0.9632	1.0000	0.9997	0.3704	4140.2	0.3705	0.9997	0.4113	0.9980
Bsa27	*B*. *safensis*	64.9	0.9970	1.0000	0.9572	1.0000	85	1.0000	0.9740	0.9972	0.0297	4273.3	0.9543	0.9998	0.9708	0.5994
Bsssu2	*B*. *subtilis* subsp. *subtilis*	66.8	0.9999	1.0000	0.9915	1.0000	163	0.3678	0.9160	0.7477	0.5509	4393.5	1.0000	0.7683	1.0000	0.1419
Bve12	*B*. *velezensis*	84.3	0.9783	0.6804	0.9992	0.8711	163	0.3678	0.9160	0.7477	0.5509	4373.8	1.0000	0.8552	1.0000	0.1886
Bve2	*B*. *velezensis*	78.2	0.9983	0.7980	1.0000	0.9553	118	0.9968	1.0000	1.0000	0.0448	4366.9	1.0000	0.8815	1.0000	0.2077
Mixture 1^f^		71.8	1.0000	1.0000	0.9979	1.0000	85	1.0000	0.9732	0.9971	0.0294	4296.1	0.9864	0.9975	0.9928	0.4842
Mixture 2^f^		77.5	1.0000	0.9700	1.0000	0.9987	169	0.5460	0.9500	0.8465	0.8607	4466.7	0.9999	0.4036	0.9996	0.0422
**Control**	**Active ingredient**^c^															
Poncho/Votivo	Clothianidin	74.0	…	0.9966	1.0000	1.0000	95	…	0.9816	1.0000	0.0071	4405.6	…	0.7082	1.0000	0.1179
	*+B*. *firmus* I-1582															
Clariva	*Pasteuria nishizawae*	64.3	0.9955	…	0.9373	1.0000	125	0.9816	…	1.0000	0.0700	4208.9	0.7082	…	0.7547	0.8979
Avicta	Abamectin	75.1	1.0000	0.9477	…	0.9961	151	0.9993	1.0000	…	0.0330	4396.3	1.0000	0.7547	…	0.1360
Untreated control	Water	68.7	1.0000	1.0000	0.9961	…	222	0.0071	0.0700	0.0330	…	4055.5	0.1179	0.8979	0.1360	…

Field trials were performed in E.V Smith and Tennessee Valley Research and Extension Center in 2015. Data collected were repeated and analyzed in SAS 9.4 using PROC GLIMMIX procedure at significant level of 0.10. Adjusted *P* values less than 0.10 indicated a significant effect. Adjusted *P* values were obtained by analyzing data according to Dunnett’s method.

^a^The LS-means are presented in the tables with adjusted *P* values for statistical differences.

^b^Bio = plant biomass including shoot fresh weight and root fresh weight (g) at 60 DAP.

^c^Activie ingredients for the nematicides Poncho/Votivo are Clothianidin plus *B*. *firmus* I-1582, Clariva is *Pasteuria nishizawae*, Avicta is Abamectin, and untreated control is water.

^d^Cyst = soybean cysts and white females in 100 cm^3^ of soil at 60 DAP.

^e^Yield = soybean yield (kg/ha) obtained at 160 DAP and adjusted to 13% moisture content.

^f^Mixture 1 = strain Bve2 + strain Bal13; Mixture 2 = Abamectin + strain Bve2 + strain Bal13.

## Discussion

*In vitro* screening of the 663 PGPR strains indicated that 13 *Bacillus* species including *B*. *altitudinis*, *B*. *aryabhattai*, *B*. *lentus*, *B*. *methylotrophicus*, *B*. *mojavensis*, *B*. *mycoides*, *B*. *pumilus*, *B*. *safensis*, *B*. *simplex*, *B*. *subtilis* subsp. *subtilis*, *B*. *toyonensis*, *B*. *velezensis*, *B*. *weihenstephanensis*, and species of *Fictibacillus* and *Paenibacillus* caused greater than 50% mortality percentage of *H*. *glycines* J2 *in vitro*. Strains of *B*. *altitudinis*, *B*. *aryabhattai*, *B*. *lentus*, *B*. *methylotrophicus*, *B*. *mojavensis*, *B*. *mycoides*, *B*. *safensis*, *B*. *simplex*, *B*. *toyonensis*, *B*. *velezensis*, *B*. *weihenstephanensis*, and strains of *Fictibacillus* were first documented in this study for antagonistic activity against *H*. *glycines*. Previously, some bacterial species have been documented to be antagonistic to *H*. *glycines*. *Bacillus megaterium* [14], *B*. *pumilus* [13, 14], *B*. *sphaericus* [15, 16], *B*. *cereus* [13], *Paenibacillus* spp. [13] were reported for their nematicidal activity on reduction of *H*. *glycines* population density in greenhouse trials. None of these studies included high throughput *in vitro* screening of biological agents to *H*. *glycines*. Our study is the first documentation of high throughput *in vitro* screening of biological control agents on efficacy to *H*. *glycines*.

*Bacillus velezensis* strain Bve2 consistently reduced *H*. *glycines* cyst numbers at 60 DAP in the greenhouse, microplot, and field trials. *Bacillus mojavensis* strain Bmo3 suppressed *H*. *glycines* cyst and total *H*. *glycines* population density under greenhouse conditions. *Bacillus safensis* strain Bsa27 and Mixture 1 (Bve2 + Bal13) reduced *H*. *glycines* cyst numbers at 60 DAP in the field trials. Individual strains of Bmo3 and Bve2 and Mixture 2 (Abamectin + Bve2 + Bal13) were previously found to reduce *M*. *incognita* eggs/g root on cotton plants in the greenhouse, microplot, and field studies [29]. This study expanded the documented nematicidal activity of the strains Bmo3 and Bve2 on *H*. *glycines*. Some studies have documented individual or mixtures of PGPR strains and/or nematicides or other agents on reduction of plant-parasitic nematode population density. Burkett-Cadena et al. [19] reported that the combination of *B*. *amyloliquefaciens* (sym. *B*. *velezensis*) strain GB99 and *B*. *subtilis* strain GB03 (BioYield, Gustafson LLC, USA) significantly reduced *Meloidogyne* spp. eggs per gram root, juvenile nematodes per cm^3^ of soil, and galls per plant on tomato. Castillo et al. [25] found that individuals strains of *B*. *firmus* GB-126 (Votivo, Bayer CropScience, Germany) and *Paecilomyces lilacinus* 251 (PL 251, Biological Control Products, South African), or the combination of *B*. *firmus* GB-126 and *P*. *lilacinus* reduced *Rotylenchulus reniformis* population density in the greenhouse, microplot, and field trials. Our results are in agreement with their studies that individual PGPR strains and mixtures have biological control potential on plant-parasitic nematodes.

*Bacillus subtilis* subsp. *subtilis* strains Bsssu2 and Bsssu3, and *B*. *velezensis* strain Bve12 increased early soybean growth including plant height and plant biomass in the greenhouse trials. *Bacillus altitudinis* strain Bal13 increased early plant growth on soybean in the greenhouse and microplot trials. Mixture 2 (Abamectin + Bve2 + Bal13) increased early plant growth in the microplot trials at 60 DAP, and also enhanced soybean yield at harvest in the field trials. Some studies have reported that individual or mixtures of PGPR strains can promote plant growth and increase yield on multiple plant hosts. Raupach and Kloepper [30] found seven PGPR seed treatments including single-strain treatments and mixtures of *B*. *pumilus* strain INR7, *Curtobacterium flaccumfaciens* strain ME1, and *B*. *subtilis* strain GB03 significantly promoted plant growth on cucumber in the field studies when methyl bromide was absent. The individual *B*. *subtilis* strain GB03 and mixture of *B*. *pumilus* strain INR7 plus *C*. *flaccumfaciens* strain ME1 promoted growth significantly on cucumber [30]. Liu et al. [31] found individual PGPR strains Bsa27 (AP7) and Bpu6 (AP18) promoted plant growth on Chinese cabbage and one strain mixture containing PGPR strains Bve12 (AP136) (*B*. *velezensis*), Bmo3 (AP209) (*B*. *mojavensis*), Lma1 (AP282) (*Lysinibacillus macroides*), Bve15 (AP305) (*B*. *velezensis*), Bsa27 (AP7) (*B*. *safensis*), Bpu6 (AP18) (*B*. *pumilus*), and Bve40 (AP218) (*B*. *velezensis*) increased shoot and root dry weights in the greenhouse test. They found that those individual strains and mixtures increased marketable yield of Chinese cabbage in the field [31]. Our study is in an agreement with previous research that individual or mixtures of PGPR strains can promote plant growth under greenhouse or field conditions and that some PGPR strains can reduce plant-parasitic nematode population density.

## Conclusions

Overall, this study indicated that *B*. *velezensis* strain Bve2, *B*. *mojavensis* strain Bmo3, and Mixture 1 (Bve2 + Bal13) have the potential to manage *H*. *glycines* on soybean. These two strains also have been found to reduce the population density of *Meloidogyne incognita* [29]. *Bacillus altitudinis* strain Bal13 and Mixture 2 (Abamectin +Bve2 + Bal13) have the ability to enhance soybean yield under field conditions. In the future, the formulation of these effective PGPR strains and mixtures should be further evaluated for the integrated management of *H*. *glycines* on soybean.

## Supporting information

S1 Table PGPR isolates effect on *Heterodera glycines* J2 mortality as compared to the industry standard biologicals Poncho/Votivo, Clariva, and chemical Temik as well as an untreated control. *In vitro* tests were performed in 96-well plates. Data collected were analyzed in SAS 9.4 using PROC GLIMMIX procedure at significant level of α ≤ 0.05. *P* value less than 0.05 indicate a significant effect. Adjusted *P* values were obtained according to Dunnett's method. The LS-means are presented in the tables with adjusted *P* values to determine statistical differences.
(XLSX)

S2 Table Effect of PGPR strains on soybean plant growth and *Heterodera glycines* population density in the greenhouse trials at 60 DAP. (XLSX)

S3 Table Effect of five PGPR strains and two mixtures on plant growth and *Heterodera glycines* population density on soybean in microplot trials at 60 DAP. (XLSX)

S4 Table Effects of five PGPR strains and two mixtures of PGPR strains on soybean plant growth and *Heterodera glycines* population density in the field trials. (XLSX)

## References

[1] Winstead NN., Skotland CB., Sasser JN. Soybean cyst nematode in North Carolina. Plant Disease Reporter. 1955; 39: 9–11.

[2] NASS. National Agricultural Statistics Service | USDA. Crop Production 2015 Summary. 46. http://www.usda.gov/nass/PUBS/TODAYRPT/cropan16.pdf. 2016.

[3] Wrather JA., Koenning SR. Effects of diseases on soybean yields in the United States 1996 to 2007. Online. Plant Health Progress. 2009 doi: 10.1094/PHP-2009-0401-01-RS

[4] Wrather A., Shannon G., Balardin R., Carregal L., Escobar R., Gupta GK., et al Effect of diseases on soybean yield in the top eight producing countries in 2006. Plant Health Progress. 2010 doi: 10.1094/PHP-2010-0125-01-RS

[5] Chen SY. Management with biological methods p. 207–242. In Biology and management of soybean cyst nematode. 2^nd^ Edition Eds. Schmitt DP., Wrather JA., and Riggs RD. Schmitt & Associates of Marceline Marceline, Missouri, USA 2004.

[6] Chen SY., Liu XZ. Control of the soybean cyst nematode by the fungi *Hirsutella rhossiliensis* and *Hirsutella minnesotensis* in greenhouse studies. Biological Control. 2005; 32: 208–219.

[7] Liu XZ., Chen SY. Parasitism of *Heterodera glycines* by *Hirsutella* spp. in Minnesota soybean fields. Biological Control. 2000; 19: 161–166.

[8] Nitao JK., Meyer SLF., Oliver JE., Schmidt WF., Chitwood DJ. Isolation of flavipin, a fungus compound antagonistic to plant-parasitic nematodes. Nematology. 2002; 4: 55–63.

[9] Tylka GL., Hussey RS., Roncadori RW. Interactions of vesicular-arbuscular mycorrhizal fungi, phosphorus, and *Heterodera glycines* on soybean. Journal of Nematology. 1991; 23: 122–133. 19283102 PMC2619123

[10] Nishizawa T. A decline phenomenon in a population of upland rice cyst nematode, *Heterodera elachista*, caused by bacterial parasite, *Pasteuria penetrans*. Journal of Nematology. 1987; 19: 546.

[11] Noel GR., Stanger BA. First report of *Pasteuria* sp. attacking *Heterodera glycines* in North America. Journal of Nematology. 1994; 26: 612–615. 19279935 PMC2619558

[12] Tian HL., Riggs RD., Crippen DL. Control of soybean cyst nematode by chitinolytic bacteria with chitin substrate. Journal of Nematology. 2000; 32: 370–376. 19270991 PMC2620463

[13] Tian H., Riggs RD. Effects of rhizobacteria on soybean cyst nematode, Heterodera glycines. Journal of Nematology. 2000; 32: 377–388. 19270992 PMC2620476

[14] Kloepper JW., Rodríguez-Kábana R., McInroy JA., Young RW. Rhizosphere bacteria antagonistic to soybean cyst (*Heterodera glycines*) and root-knot (*Meloidogyne incognita*) nematodes: identification by fatty acid analysis and frequency of biological control activity. Plant and Soil. 1992; 139: 75–84.

[15] Sharma RD. Efficiency of *Bacillus* spp. toxins to control *Heterodera glycines* on soybean. Nematologia Brasileira. 1995; 19: 72–80.

[16] Sharma RD., Gomes AC. Effect of *Bacillus* spp. toxins on oviposition and juvenile hatching of *Heterodera glycines*. Nematologia Brasileira. 1996; 20: 53–62.

[17] Kloepper JW., Ryu CM., Zhang SA. Induced systemic resistance and promotion of plant growth by *Bacillus* spp. Phytopathology. 2004; 94: 1259–1266. doi: 10.1094/PHYTO.2004.94.11.1259 18944464 10.1094/PHYTO.2004.94.11.1259

[18] Keren-Zur M., Antonov J., Bercovitz A., Feldman K., Husid A., Kenan G., et al. Bacillus firmus formulations for the safe control of root-knot nematodes. In: Proceedings of the Brighton Crop Protection Conference on Pests and Diseases. 2000; Vol. 2A. 47–52.

[19] Burkett-Cadena M., Kokalis-Burelle N., Lawrence KS., Van Santen E., Kloepper JW. Suppressiveness of root-knot nematodes mediated by rhizobacteria. Biological Control. 2008; 47: 55–59.

[20] Hallmann J., Davies KG., Sikora RA. Biological control using microbial pathogens, endophytes and antagonists p. 380–411. In Root-knot nematode. Eds. Perry RN., Moens M., Starr JL. CAB International Wallingford, UK 2009.

[21] Wilson MJ., Jackson TA. Progress in the commercialization of bionematicides. BioControl. 2013; 58: 715–722.

[22] Askary TH. Limitation, research needs, and future prospects p. 446–454. In Biocontrol agents of phytonematodes. Eds. Askary TH., and Martinelli PRP. CAB International Wallingford, OX, UK 2015.

[23] Riggs RD., Schmitt DP. Optimization of the *Heterodera glycines* race test procedure. Journal of Nematology. 1991; 23: 149–154. 19283105 PMC2619141

[24] Jenkins W. A rapid centrifugal-floatition technique for separating nematodes from soil. Plant Disease Report. 1964; 48: 692.

[25] Castillo JD., Lawrence KS., Kloepper JW. Biocontrol of the reniform nematode by *Bacillus firmus* GB-126 and *Paecilomyces lilacinus* 251 on cotton. Plant Disease. 2013; 97: 967–976. 10.1094/PDIS-10-12-0978-RE 30722537

[26] Xiang N., Lawrence KS., Kloepper JW., Mcinroy JA. In vitro screening of biological control agents on Meloidogyne incognita. Proceedings of the 2014 Beltwide Cotton Conference. Vol 1:258–260. National Cotton Council of America, Memphis, TN. 2014.

[27] Xiang N., Lawrence KS. Optimization of *in vitro* techniques for distinguishing between live and dead second stage juveniles of *Heterodera glycines* and *Meloidogyne incognita*. Plos One. 2016; 11: e0154818 doi: 10.1371/journal.pone.0154818 27144277 10.1371/journal.pone.0154818 PMC4856281

[28] Schrimsher DW., Lawrence KS., Sikkens RB., Weaver DB. Nematicides enhance growth and yield of *Rotylenchulus reniformis* resistant cotton genotypes. Journal of Nematology. 2014; 46: 365–375. 25580030 PMC4284089

[29] Xiang N., Lawrence KS., Kloepper JW., Donald PA, McInroy JA., Lawrence GW. Biological control of Meloidogyne incognita by spore-forming plant growth-promoting rhizobacteria on cotton. Plant Disease. 2017 101: 774–784. https://doi.org/10.1094/PDIS-09-16-1369-RE. 10.1094/PDIS-09-16-1369-RE 30678579

[30] Raupach GS., Kloepper JW. Biocontrol of cucumber diseases in the field by plant growth-promoting rhizobacteria with and without methyl bromide fumigation. Plant Disease. 2000; 84: 1073–1075. 10.1094/PDIS.2000.84.10.1073 30831895

[31] Liu K., Garrett C., Fadamiro H., Kloepper JW. Induction of systemic resistance in Chinese cabbage against black rot by plant growth-promoting rhizobacteria. Biological Control. 2016; 99: 8–13.

